# Cost-Effectiveness of Thrombolytic Therapy, Compared with Anticoagulants Therapy in the Treatment of Acute Myocardial Infarction in Albania

**DOI:** 10.3889/oamjms.2015.060

**Published:** 2015-05-28

**Authors:** Mirvete Rama, Mirela Miraci, Idriz Balla, Ela Petrela, Ledjan Malaj, Anjeza Koleci

**Affiliations:** 1 *Pharmaceutical Service “Mother Teresa” Hospital, Tirana, Albania*; 2 *Medicine University, Faculty of Pharmacy, Tirana, Albania*; 3 *University Hospital Center “Mother Theresa”, Tirana, Albania*; 4 *Medicine University, Public Health Faculty, Tirana, Albania*; 5 *Bayer Representation Office, Tirana, Albania*

**Keywords:** Acute Myocardial Infarction, Cost-effectiveness, Thrombolytics, Streptokinase (SK), Reteplase(r-Pa), anticoagulants

## Abstract

**AIM:**

The study aim is to evaluate the cost-effectiveness of thrombolytic treatment in acute MI comparing with anticoagulants therapy and between each other thrombolytic (SK, r-Pa).

**MATERIAL AND METHODS:**

We used a prospective registry of all patients admitted for acute myocardial infarction in intensive care units in Tirana. The average drugs cost was calculated for the hospitalization period in Albanian money (ALL). Survival and life quality were estimated by phone contact 1 year after acute MI.

**RESULTS:**

Anticoagulant group cost is 23865.3 ALL (170.5€), SK group cost is 54148.63 ALL (386.7€), r-Pa group has a cost of 92184.90 ALL (658.5€). In the group treated with SK the hospital survival is 100%, while in the control group 88.8%. Reteplase group has a lower period of stay in hospital than SK group 13.04 days vs. 17.97 days, mean age in group treated with r-Pa is 64.29 ± 10.03 approximate with anticoagulant group mean age 64.17 ± 11.08; differ significantly with SK group mean age 56.75 ± 10.04. Survival after 1 year was 96.4% for r-Pa and 96.9% SK.

**CONCLUSIONS:**

SK and r-Pa are successful thrombolytics with high effectiveness. It is gained a higher survival with the thrombolytic treatments. Reteplase is well tolerated in older patients than SK, is easier to apply than Streptokinase, but has higher cost.

## Introduction

Acute myocardial infarction (AMI) remains one of the major causes of morbidity and mortality in the world, caused by the closure of coronary arteries due to thrombus. Deaths with coronary heart disease cover about 7 million people (about 30% of mortality, according to the WHO). In Europe around 2 million people die annually from cardiovascular diseases. From literature also it is noted that AMI appears almost twice more in men than in women (10% vs. 6%) [1]. The purpose of the thrombolytic therapy is the reduction of mortality after myocardial infarction and improvement of life quality to patients with acute MI. Thrombolytic therapy has been a major breakthrough in the management of acute myocardial infarction. This treatment works by merging thrombus formed in arteries and reaching a circulation of blood to the arteries in order to avoid death and improve survival [2]. Thrombolytic drugs are most effective if administered immediately after the determination of first symptoms of the AMI. The benefit of thrombolytics use is higher when they apply within the first 6-12 hours of symptoms [3]. These drugs are usually used in combination with anticoagulants such as heparin, fraksiparina, calciparina, dalteparina, enoxaparina [4].

Streptokinase belongs to the first generation thrombolytic group. SK disadvantage is that it has little specificity for fibrin, increases the risk of allergic reactions and it has a low biological half-life [5].

Reteplase is the newest thrombolytic agent (generate III), used in the treatment of acute MI, with longer biological half-life of advantage [6].

However a numerous studies (GUSTO, INJECT) showed no significant changes play regarding hospital survival between these thrombolytics used to treat acute MI. [7, 8]. Also in others studies like RAPID-II and GUSTO-III is proven effectiveness of these thrombolytics used and results after a period of one year showed no significant differences between the two treatments [9, 11].

The purpose of this study is: - to compare survival between the two thrombolytics; Reteplase vs. streptokinase; - to compare survival between groups who have used anticoagulants vs thrombolytic treatment of AMI; and - to estimate the cost of treatment of acute myocardial infarction, respectively in each group.

The aim of this study is to evaluate thrombolytic cost - effectiveness therapy in acute myocardial infarction in intensive care hospital in Tirana as a better choice to reduce mortality, calculation of the hospital treatment cost and finally finding out the cost- effective treatment. This cost- effectiveness analyses will be a novel solution for hospital treatments here in Albanian hospitals where cardiology intensive care is offered.

## Material and Methods

We used a prospective registry of all patients admitted for acute myocardial infarction in intensive care units in Tirana. The study included 332 patients with acute myocardial infarction (MI), which were in- hospital treatment, at Intensive Care Clinic of the University Hospital Centre, “Mother Theresa” Tirana, in the period 2010-2012. 120 patients (28females and 92males) received thrombolytic therapy (streptokinase or reteplase), the rest served as control group (212 patients; 53 females and 159 males) and received standard therapy with anticoagulants: fraxiparine, Plavix, aspirin, heparin. The patients in the control group were treated with standard anticoagulants therapy in 2010-2011, because there no thrombolytic drug in our hospital during this period of time or it was in small amounts. These patients were diagnosed as the same as the thrombolytic group with AMI. In the group of patients receiving r-PA took part 56 patients. 64 patients were part of SK treatment. We compared the thrombolytic treatment vs. anticoagulant and also SK with r-Pa.

It is calculated the average drugs cost for each patient for the period of stay in hospital. Survivals, mortality and quality of life were defined through interviews with telephone contact to patients 1 year after the acute MI.

Quantitative variables continuous and discrete were used, as well as qualitative variables. Continuous variables were presented in the average value and standard deviation, and discrete variables in absolute value and percentage. It is used student test for significant differences in hospital stay and the confidence interval for comparisons between drugs in the two groups. To compare the situation of patients after treatment was used Hi-Square test. There were considered significant values of P ≤ 0.05 (P ≤ 5%)

Data analysis was performed with SPSS statistical package, version 18.

## Results

The study enrolled a total of 388 patients with Acute Myocardial Infarction that were hospitalized at Intensive Care Clinic, in the period 2009-2012. After the data were collected; the results were presented with Tables and Figures.

Table 1 shows that control group has a mean age greater than thrombolytic group (64.2 ± 11.8 vs. 56.7 ± 10.3). There are significant difference between two groups p ≤ 0.005. In this table is compared control group with thrombolytic group taken together as one.

**Table 1:** Summary data of each group

Variables	Total population n= 332	Gr. with SK n=64	Gr. With r-Pa n=56	Gr. With anticoag n=212	p-value
Age (in years)		56.03	64.29	64.17	0.001

Gender				
M		48	44	159
f		16	12	53	

Survival		96.9%	96.4%	88.8%	<0.005

**Table 2:** Presentation of age in the (SK+ r-Pa) group and control group

					Std. Error
	Group	N	Mean	Std. Deviation	Mean

Age-year	SK and r-Pa	120	56.7479	10.03994	0.92036
	Anticoagulants	212	64.1745	11.08745	0.76149

**Figure 1:**
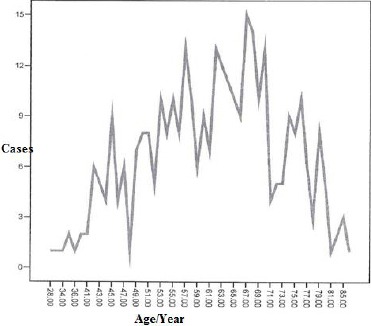
*Number of cases with AMI by age*.

It is showed that the largest number of cases with acute MI is concentrated in the age group 60-70 years. Acute MI is less common in extreme ages 28-34 and 80-85 years old (Fig.1).

**Figure 2:**
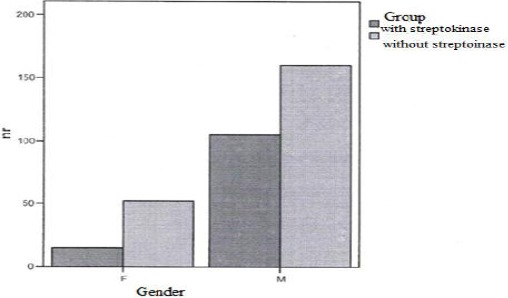
*Number of patients according the gender and groups*.

Figure 2 shows clearly that acute MI affects males more than females. There are more males with acute MI in both groups than females.

**Table 3:** It shows the average age in thrombolytic groups

	Medicine	Nr of cases	Average	SD	SE	t	Df	Value of p
Age	Reteplase	56	64.29	10.03	1.89	3	58	0.001

	Streptokinase	64	56.03	8.71	1.54			

There were compared the thrombolytics between each other (tab.3). The average age in the group with r-Pa (64.29 ± 3.10) is higher than the average age of the SK group (56.75 ± 4.10), P = 0.001. This shows that reteplase is tolerable even in higher ages.

Table 4 shows hospital survival in each thrombolytic group (r-Pa), (SK) and anticoagulant group. There is only one dead patient at SK group and so it is at r-Pa, while at anticoagulant there are 10 dead patients. Survival rate at r-Pa group is 96.4%, at SK group is 96.9%, at anticoagulant Group 95.3%

**Table 4:** Survival comparison between each group period at the end of the hospital treatment

Hospital survival in r-Pa, SK and anticoagulant groups		Reteplase (N=56)	Streptokinase (N=64)	Anticoagulant N=212	Total (N=332)
Patients after treatment	Dead	1	1	10	12

		3.6%	3.1%	4.7	3.01%

	Improved	55	63	202	320
		96.4%	96.9%	95.3%	96.7%

**Table 5:** Comparison between hospital stay periods of time in each group

	Treatment	Nr of cases	Mean	SD	SE	t	Df	Value p
Hospital stay period	Reteplase	56	13.04	5.92	1.12	-3	58	0.010

	Streptokinase	64	17.97	8.14	1.44			

	Anticoagulant	120	16.02	8.3	1.20			

The group treated with r-Pa has an average stay 13.04 days/patient in the hospital with SD 5.92. The group treated with SK has an average stay17.97 days/patient in hospital and SD 8.14 and anticoagulant. Group has an average hospital stay of 16.02 days, where P=0.01.

Table 6 shows the situation 1 year after the treatment, where there are no significant differences between the two treatments with thrombolytics.

**Table 6:** Comparison after one year of acute MI treatment

		Treatment		Total
		Reteplase (N=56)	Streptokinase (N=64)
Situation 1 year after treatment	Dead	1 (3.6)	1 (3.2)	2 (3.3)

	Worse	0 (0.0)	4 (6.4)	4 (3.3)

	Good	41 (71.4)	35 (54.8)	76 (61.7)

	Very good	14 (25.0)	24 (38.6)	38 (31.7)

The cost in r-Pa group is 658.5€, in SK group 386.8€ and in anticoagulant group it is 207.4€ Treatment with r-Pa has a cost of 38036.27 ALL (271.7 €) higher than treatment with SK, and 451.1€, P = 0.001, otherwise anticoagulant group has a lower treatment cost than both other groups.

**Table 7:** It shows the treatment cost in each group

	Treatment	Nr of cases	Mean	SD	SE	t	D f	Value p
Total cost	Reteplase	56	92184.90	11191.19	2114.94	12	58	<0.001

	Streptokinase	64	54148.63	13693.78	2420.74			

	Anticoagulant	212	29030.85	18066.39	2695.34			

## Discussions

Acute Myocardial Infarction is shown in males people more than in females [10]. In our study 75.6% of cases with AMI are males and only24.4% are females. The available data from clinical trials have shown that reteplase acts faster than SK in patients with acute MI [12]. In our study we assessed both thrombolytic used, and the group with anticoagulants. The thrombolytic therapy is more effective than anticoagulants therapy for AMI [2]. Assessing in- hospital mortality in all groups, we evaluated the advantages of thrombolytics compared with standard anticoagulant therapy. The mortality rate was 3.6% in r-Pa group, 3.1% in SK group and 4.7% in anticoagulant group. From numerous studies it was observed that between two thrombolytics SK and r-PA (Inject study) [13] there were no significant differences, so the hospital survival in our study is approximate 96.4% in the group with r-Pa and 96.9% in the group with SK. While in the group treated with anticoagulants there are 10 cases of death and survival is 95.3%.

From another study has found that r-Pa has a much higher treatment cost than SK (GUSTO-I study) [14]. Likewise, in our study r-Pa has higher costs than SK and anticoagulants (€ 685.5 vs. 386.8 €, 207.4€). The high treatment cost of reteplase has to be evaluated in further studies in which must be defined the efficiency and the cost (QALY/life saved). Even though there are not direct comparing studies between reteplase and streptokinase regarding survival rate one year after thrombolytic treatment of acute MI [16], in this study it is shown the equivalence in survival and no significant differences between two thrombolytics.

In conclusion, Streptokinase and Reteplase are successful thrombolytics in treatment of acute myocardial infarction, with high efficiency comparing with anticoagulants therapy. It is obtained a greater survival with thrombolytic treatment compared with the control group treated with anticoagulants’ therapy. Reteplase is more acceptable to older ages than SK. Reteplase is more easily applicable than SK. r-Pa has a higher treatment cost then SK, however, it is an added value in the range of thrombolytics used in the treatment of AMI.
